# Labeling Pathological Tau: An Important Quest for the Unknown

**DOI:** 10.3389/fnins.2016.00253

**Published:** 2016-06-06

**Authors:** Irving E. Vega

**Affiliations:** Department of Translational Science and Molecular Medicine, College of Human Medicine, Michigan State UniversityGrand Rapids, MI, USA

**Keywords:** tau, brain imaging, tauopathy, Alzheimer's disease, fluorescent fibrillary tau dye

Biomedical research directed to understand the pathophysiology of Alzheimer's disease (AD) has contributed to the characterization of two pathological hallmarks of this terrible disease, namely senile plaques and neurofibrillary tangles. Neuropathological studies indicated that the level of accumulation of these pathological lesions correlate with the severity of the clinical presentation (Braak and Braak, 1991; Hyman et al., 2012). Senile plaques are formed by the accumulation of Aβ peptides derived from the proteolysis of the Amyloid Precursor Protein, while neurofibrillary tangles are ultrastructures predominantly compose of aberrantly phosphorylated tau proteins. Previous studies indicated that reduction of Aβ peptides and increase of phosphorylated tau peptides in the cerebrospinal fluid (CSF) serve as biomarkers to confirm the diagnosis of probable AD (Olsson et al., 2016). Despite the detected correlation, questions about the sensitivity of the assay and temporal disconnect with the genesis of pathological tau in the brain, as well as the invasive nature of a lumbar puncture, makes CSF analysis a rather unlikely approach for mainstream clinical laboratory testing. Alternatively, serum and/or plasma are routinely used for clinical diagnosis purposes. However, the specificity of putative biomarkers has not reach a desirable level of certainty for the diagnosis of AD (Olsson et al., 2016). Therefore, due to the low invasiveness and promising results already obtained, most research efforts have been re-directed to develop selective molecules that facilitate the imaging of pathological proteins in living neurons and individuals.

The knowledge gained from neuropathological studies of AD cases has been used as the basis to direct the development of molecules that are able to specifically interact with Aβ or tau oligomers, in brain regions of interest in living individuals. The selection of these brain regions of interest is based on the hierarchical and sequential appearance of pathological lesions in postmortem tissue and its correlation with functional impairments observed in living individuals (Braak and Braak, 1991; Hyman et al., 2012). Although different studies showed that the detection of Aβ pathology precede the detection of pathological tau, it is the accumulation of tau aggregates at specific brain regions that directly correlates with executive dysfunction and cognitive impairment in AD (Arriagada et al., 1992; Jack et al., 2013). Hence, different molecules have been developed in the quest to selectively visualize and study the accumulation of pathological tau proteins.

Brelstaff et al. (2015) demonstrated that pentameric formyl thiophene acetic acid (pFTAA) is able to bind filamentous tau proteins in living neurons. pFTAA is a fluorescence dye that selectively binds to beta-sheets conformations characteristic of amyloid proteins, including Aβ oligomers and fibrilar tau. In this study, pFTAA was injected intravenously via the tail vein of transgenic mice overexpressing the human tau-P301S mutant gene (Brelstaff et al., 2015). The mice were euthanized after 48 h and the brain stained with antibodies that are known to detected pathological tau (e.g., MC1, AT100). The results showed that peripherally injected pFTAA selectively labeled neurons that were positive for the presence of pathological tau, demonstrating the blood brain barrier permeability of pFTAA and its uptake by living neurons. Consistently, pFTAA also label pathological tau formed in cultured dorsal root ganglion neurons from htau-P301S transgenic mice. They also demonstrated that pFTAA positive neurons in culture had a shorter survival rate than pFTAA negative neurons (Brelstaff et al., 2015). Thus, this study demonstrated, for the first time, that pFTAA detects pathological tau in living neurons, becoming a valuable tool to assess compounds that target the aggregation and/or promote the disaggregation of pathological tau in cultured neurons, including iPSC-derived neurons from tauopathy patients. Additionally, pFTAA labeling may facilitate the study of molecular mechanisms that contribute to pathological tau aggregation in cultured neurons. Although, this study represents an important progress in the development of molecular tools to study the formation and metabolism of aggregated tau, we need to take in consideration the limitations of assessing the formation of toxic forms of tau in cultured cells. In addition, many important questions regarding tau-mediated neurodegeneration remain unanswered.

The main question in AD and other tauopathies that remain unanswered is the definition of pathological tau. For example, pFTAA staining indicates that the presence of fibrillar tau is associated with reduced survival of cultured neuron. However, due to the temporal cell death process of pFTAA positive cells, it is difficult to differentiate between the toxic effect that early stages of tau oligomerization and later forms of fibrillar tau could exert to the cell. In this regard, we should ask: what specific aberrant tau structure is toxic to neurons? Is there a temporal accumulation of different tau structures or aggregate species that mediate distinct pathological signals? Are neuropathological hallmarks (neurofibrillary tangles) a cause or consequence of the pathophysiological process? Is the lack of detectable tau aggregates enough evidence to determine that a specific brain region is resilient to tauopathy or that the brain is free of toxic tau? Perhaps, the temporal characterization of different tau species using conformation-specific anti-tau antibodies and labeling molecules, such as pFTAA or tau-specific PET radiotracers (Shimojo et al., 2015), could provide insights about the evolution of toxic tau.

Johnson et al. (2016) and Schöll et al. (2016) got closer, using the positron emission tomography (PET) tau-ligand AV-1451, to achieving the ultimate goal of performing brain imaging to track the progression of tau pathology in living individuals. However, the correlation of tau levels and cognitive decline suggests that patients already experienced significant brain damage before detection of pathological tau. Moreover, it is not clear if detection of AV-1451 in “healthy aging brain” represents a pre-clinical stage or a normal by-product of tau metabolism. Although the optimization of molecules that selectively detect specific tau pathological species will be extremely beneficial for diagnosis and assessment of therapeutic interventions, research effort should also be directed to understand molecular signals that could be used to detect cellular stress before pathological aggregates are detected. Therefore, further studies that contribute to define the structural characteristics and temporal accumulation of pathological tau species and the cellular response to these species will contribute to the development of better diagnostic tools.

## Author contributions

IEV revised the literature and wrote the manuscript.

### Conflict of interest statement

The author declares that the research was conducted in the absence of any commercial or financial relationships that could be construed as a potential conflict of interest.
